# Supplementary material for: Behavioural and socio-ecological factors that influence access and utilisation of health services by young people living in rural KwaZulu-Natal, South Africa: Implications for intervention
Source: PLoS One. 2020 Apr 14;15(4):e0231080. doi: 10.1371/journal.pone.0231080 (PMC7156071; doi:10.1371/journal.pone.0231080)
Supplement: Attachment — Submitted filename: Response to reviewers.docx [file pone.0231080.s001.docx]

**Rachel A. Annunziato, Ph.D.**

**Academic Editor**

**PLOS ONE**

Date: 29 February 2020

Dear Professor Rachel A. Annunziato,

**Manuscript title: Behavioural and socio-ecological factors that influence access and utilisation of health services by young people living in rural KwaZulu-Natal, South Africa: Implications for intervention.**

We would like to thank you for the consideration given to our manuscript as well as the reviewers for their time in giving comments to improve this work.

We have included the reviewers’ comments as well as the editor ‘suggestions immediately after this letter and responded to them individually, indicating how we addressed each point raised. The revisions have been approved by all the authors.

We hope the revised manuscript now meets the required standards of PLOS One and we thank you for your interest in our research.

**Kind regards**

**Nothando Ngwenya**

1. Please ensure that your manuscript meets PLOS ONE's style requirements, including those for file naming. The PLOS ONE style templates can be found at http://www.plosone.org/attachments/PLOSOne_formatting_sample_main_body.pdf and <http://www.plosone.org/attachments/PLOSOne_formatting_sample_title_authors_affiliations.pdf>

Response: We have followed PLOS ONE’s publishing style requirements and checked all formatting against the templates provided.

2. Please change "female” or "male", when used as a noun, to "woman”, "man", "boy", "girl" or other such terms, as appropriate.

Response: Thank you for the suggestion. We have made the necessary changes in the instances where ‘female’ or ‘male’ was used as a noun and removed other instances for readability.

3. In the ethics statement in the Methods and online submission information, please ensure that you have specified, both for participant consent and parental consent, (1) whether consent was informed and (2) what type you obtained (for instance, written or verbal, and if verbal, how it was documented and witnessed).

Response: All the consent was informed, as well as assent for minors. Only written informed consent was obtained from all participants as well as from parents in the case of minors under the age of 18 years old. Verbal consent was not used in this research.

4. We note that you have indicated that data from this study are available upon request. PLOS only allows data to be available upon request if there are legal or ethical restrictions on sharing data publicly. For more information on unacceptable data access restrictions, please see <http://journals.plos.org/plosone/s/data-availability#loc-unacceptable-data-access-restrictions>.

Response: There are ethical restrictions on sharing these data publicly due to data containing potentially identifying participant information.

5. If there are ethical or legal restrictions on sharing a de-identified data set, please explain them in detail (e.g., data contain potentially sensitive information, data are owned by a third-party organization, etc.) and who has imposed them (e.g., an ethics committee). Please also provide contact information for a data access committee, ethics committee, or other institutional body to which data requests may be sent.

Response: We have indicated this in the Data availability statement, “*Data cannot be shared publicly because of confidentiality and potential breach as the data contains potentially identifying participant information. Data are available from the AHRI Research Data Management committee (contact via RDMServiceDesk@ahri.org) for researchers who meet the criteria for access to confidential data.*”

6. If there are no restrictions, please upload the minimal anonymized data set necessary to replicate your study findings as either Supporting Information files or to a stable, public repository and provide us with the relevant URLs, DOIs, or accession numbers. For a list of acceptable repositories, please see <http://journals.plos.org/plosone/s/data-availability#loc-recommended-repositories>.

Response: “Data cannot be shared publicly because of confidentiality and potential breach as the data contains potentially identifying participant information. Data are available from the AHRI Research Data Management committee (contact via RDMServiceDesk@ahri.org) for researchers who meet the criteria for access to confidential data.”

7. We note you have included a table to which you do not refer in the text of your manuscript. Please ensure that you refer to Table 1 in your text; if accepted, production will need this reference to link the reader to the Table.

Response: Thank you for this suggestion and we have updated the participants and procedures section pg 8, lines 148 – 150 to help make an easier reference to the Table.

Review Comments to the Author

8. Reviewer #1: The manuscript is technically sound scientific research with robust data points that support the conclusions. The use of both quantitative and qualitative data presents a comprehensive exploration of health service utilization by young people in rural KwaZulu-Natal, South Africa.

Response: We thank the reviewer for their comment and appreciation of this manuscript being technically sound scientific research.

9. Statistical analysis has been performed appropriately. However, I was unable to locate the summarizing statistics in Figures 1-3. These figures did not seem to appear in the manuscript and would have greatly contributed to my understanding of the statistical analysis and data visualization tools used.

Response: Figures 1-3 were a visualisation of the theoretical frameworks that were applied to assist with the data analysis as well as the intersection and interaction of the theories used. We found this useful in describing the pathway we took in conducting the analysis and shows the analytical framework used to identify indicators, constructs and characteristics associated with young people’s behaviour in our research community.

10. Language in manuscript is clear, correct, and unambiguous. Results are presented in an intelligible fashion that is also accessible to non-experts.

Response: We thank the reviewer for this positive comment as we tried to ensure that the content, especially that of the theoretical underpinning was accessible to non-experts.
